# Circulating Histones in Sepsis: Potential Outcome Predictors and Therapeutic Targets

**DOI:** 10.3389/fimmu.2021.650184

**Published:** 2021-03-24

**Authors:** Yupei Li, Dingyuan Wan, Xinyao Luo, Tao Song, Yiran Wang, Qiao Yu, Luojia Jiang, Ruoxi Liao, Weifeng Zhao, Baihai Su

**Affiliations:** ^1^Department of Nephrology of West China Hospital, Institute for Disaster Management and Reconstruction, Sichuan University, Chengdu, China; ^2^Department of Emergency Medicine of West China Hospital, Disaster Medical Center, Sichuan University, Chengdu, China; ^3^Med-X Center for Materials, Sichuan University, Chengdu, China; ^4^West China School of Medicine, Sichuan University, Chengdu, China; ^5^College of Polymer Science and Engineering, State Key Laboratory of Polymer Materials Engineering, Sichuan University, Chengdu, China

**Keywords:** sepsis, heparin, inflammation, survival, cytotoxicity, circulating histones, coagulation

## Abstract

Sepsis is defined as a life-threatening organ dysfunction caused by a dysregulated host response to infection and is associated with high morbidity and mortality. Circulating histones (CHs), a group of damage-associated molecular pattern molecules mainly derived from neutrophil extracellular traps, play a crucial role in sepsis by mediating inflammation response, organ injury and death through Toll-like receptors or inflammasome pathways. Herein, we first elucidate the molecular mechanisms of histone-induced inflammation amplification, endothelium injury and cascade coagulation activation, and discuss the close correlation between elevated level of CHs and disease severity as well as mortality in patients with sepsis. Furthermore, current state-of-the-art on anti-histone therapy with antibodies, histone-binding proteins (namely recombinant thrombomodulin and activated protein C), and heparin is summarized to propose promising approaches for sepsis treatment.

## Introduction

Sepsis is a dysregulated host response secondary to infection, which leads to life-threatening multiple organ dysfunction syndrome (MODS) and subsequent death (1). It is estimated that 31.5 million people are affected by sepsis, with 5.3 million deaths worldwide annually (2). The latest nationwide epidemiologic study in China also indicated that the frequency of sepsis in the intensive care units (ICU) was 20.6 cases per 100 ICU admissions with a high 90-day mortality of 35.5% (3). Current clinical management of sepsis mainly includes broad-spectrum antibiotics therapy, fluid resuscitation, vasopressor administration, and organ support (4). However, these conventional therapies overlook the immunopathological nature of sepsis and thus fail to further improve the survival rates of patients with severe sepsis and septic shock (5, 6). Consequently, emphasis has recently been laid on identifying novel therapeutic targets and prognostic biomarkers for sepsis treatment to achieve proper and timely immunomodulation of this dysregulated immune response (7, 8).

Histones, a group of positively charged nucleoproteins, participate in packing DNA into chromatin and regulating gene expression (9). When tissues are exposed to hostile microenvironment, histones are released into the blood circulation and act as damage-associated molecular pattern (DAMP) molecules, which mediate inflammation response, organ injury and death through activating Toll-like receptors (TLR) and NLRP3 inflammasome pathways (10, 11). Emerging studies further indicate that high concentrations of circulating histones (CHs) are significantly associated with disease severity and mortality (12). In this review, we set out to elucidate the detailed immunopathological role of CHs in sepsis and discuss the feasibility of utilizing CHs as potential biomarkers for outcome prediction of sepsis. Furthermore, potential therapeutic agents for histone neutralization are summarized to propose novel promising therapies for sepsis management.

## Immunopathological Role of Circulating Histones in Sepsis

### Basics of Circulating Histones

The high-resolution structures of histones have been identified by structural biology techniques during the past few decades. Histones, basic subunits of chromatin, are categorized into core histones (H2A, H2B, H3, and H4) and linker histones (H1 and H5) (13). Each histone has its N-terminal tail rich in lysine and arginine residues that extend out from the core structure, which accounts for the basic nature of the histone proteins (11). In physiological conditions, histones construct nucleosomes and participate in DNA transcription, replication, and repair (12, 13). However, they can also function as endogenous danger signals or DAMPs when they translocate from the nucleus to blood circulation under sepsis, trauma, or pancreatitis (14–18).

Release of histone into extracellular space requires the rupture of the nuclear and plasma cell membrane like necrosis that occurs extensively in acute organ injury (11). Histones are also present in the surrounding extracellular space after other forms of regulated necrosis including necroptosis, pyroptosis and ferroptosis and are also expressed at the surface of apoptotic cells (11, 19). Likewise, NETosis/ETosis, a regulated form of necrosis that is restricted to immune cells like neutrophils (NETosis) and other granulocytes or macrophages (ETosis), also contributes to the formation of CHs (20–24). In sepsis, the decondensed chromosome networks, termed as neutrophil extracellular traps (NETs), are mainly formed by neutrophil protrusions after stimulations of interleukin (IL)-8, lipopolysaccharide (LPS), TNF-α, complement, high hemodynamic forces and cold-inducible RNA-binding protein (11, 25–28). Growing evidence have suggested that activation of TLR2/4 as well as complement play a significant role in initiating NETosis and mediating dysregulated innate immune response, forming a vicious cycle to cause subsequent tissue injury and organ dysfunction (29–31). Besides, activation of both NOX-dependent and -independent pathway, which significantly facilitates NETosis, can also reduce histones’ adhesive ability to the NETs and then lead to the release of histones into blood circulation (32).

### Immunopathological Roles of Circulating Histones in Sepsis

**Figure 1:**
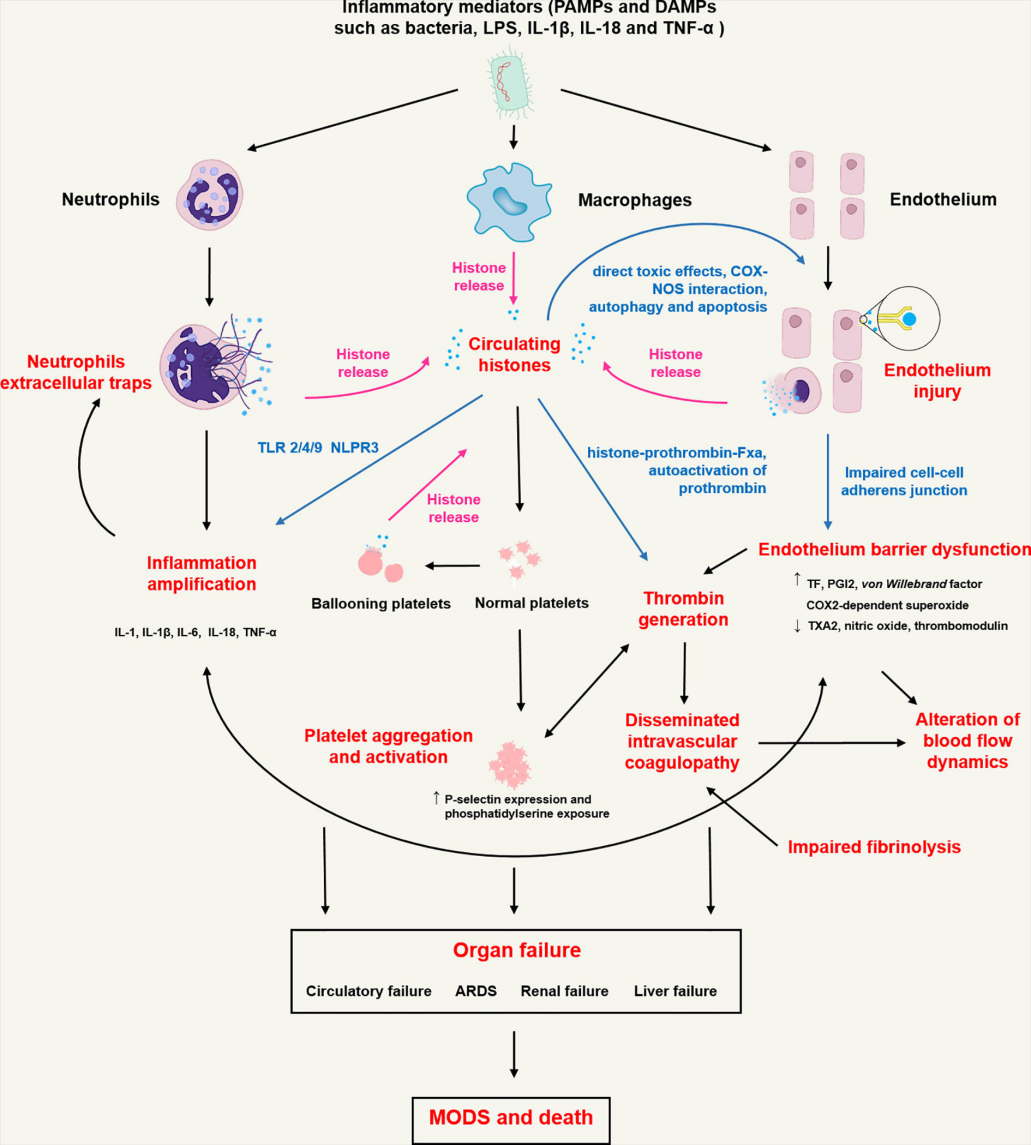
Proposed immunopathological roles of circulating histones in sepsis. In sepsis, inflammatory mediators including pathogen associated molecule patterns (PAMPs, namely bacteria and LPS etc.) and damage associated molecule patterns (DAMPs, namely IL-18, TNF-α and high mobility group box 1 etc.) activate innate immune cells (neutrophils and macrophages) and endothelial cells through pattern recognition receptors (toll-like receptors, C-type lectin receptors, and NOD-like receptors), initiating transcription of type I interferons and proinflammatory cytokines and triggering highly inflammatory programmed cell death such as NETosis/ETosis, necroptosis, necrosis and pyroptosis. Rupture of the plasma cell membrane of these cells contributes to the release of histones into extracellular spaces. Histones trigger innate immunity by activating Toll-like receptor (TLR) 2/4/9 or NLPR3 inflammasome pathways, leading to the release of pro-inflammatory cytokines (IL-1, IL-1β, IL-6, IL-18, TNF-α etc.) that not only cause neutrophils to release extracellular traps in return but also upregulate tissue factor (TF) expression by blood monocytes. Histones bind to endothelium cells and cause cell permeabilization, calcium influx and endothelium injury. Endothelium injury further induces barrier dysfunction, leakage of plasma-protein-rich fluid into the tissues, alteration of blood flow dynamics, recruitment and activation of circulating leucocytes and release of excessive cytokines, which are characterized by increased TF, prostacyclin (PGI2) and superoxide expression, decreased thromboxane A2 (TXA2) and nitric oxide release. Increased TF can trigger the activation of prothrombin through extrinsic coagulation pathway and finally cause disseminated intravenous coagulation (DIC). Besides, histones can directly activate thrombin formation by binding to prothrombin fragments 1 and 2 to form histone-prothrombin-FXa complex. Histones also induce platelet aggregation, activation and subsequent platelet-dependent thrombin formation through TLR2 and TLR4 pathways. Finally, dysregulated inflammation response, impaired endothelial barrier, immunothrombosis and DIC collectively cause multiple organ dysfunction (MODS) including circulatory failure, acute respiratory distress syndrome (ARDS), renal failure and liver failure, resulting in death in most severe form of sepsis.

As with many mediators of innate immunity, histones have been shown to trigger inflammatory responses, endothelium injury, and cascade coagulation activation. The proposed immunopathological roles of CHs in sepsis are shown in **Figure 1**. Detailed pathophysiological processes involved in sepsis are discussed below.

#### Inflammation Amplification

##### TLR-Dependent Pro-Inflammatory Cytokine/Chemokine Release

The involvement of TLR pathway in sepsis has been clearly pointed out although detailed mechanism remains unexplored (4, 33). TLRs are type I membrane proteins used for recognizing pathogen-associated-molecule-patterns (PAMPs) as well as DAMPs and initiating host innate immune response. In sepsis, CHs interact with the ectodomain of TLR2/4/9 and spark MyD88, which recruits IL-1 receptor-associated kinase family with tumor necrosis factor (TNF) receptor-associated factor 6, activates the NF-κB pathway and eventually leads to a significant rise in TNF-α and IL-6 level (11, 34, 35). Subsequent results range from single organ injury to severe sepsis (34, 36). TLR4 also motivates Toll/IL-1-receptor-domain-containing adaptor protein resulting in the up-regulation of interferon-beta (37). These pro-inflammatory cytokines soon activate innate and adaptive immune system, form a vicious circle, and induce sepsis. Moreover, release of histone H4 in sites of infection or inflammation may potentiate neutrophil activation in return and promote additional inflammatory responses that is characterized by sustained rise in neutrophil intracellular calcium, respiratory burst activation and degranulation (38). Histones specifically target monocytes in human blood, which evokes the mobilization of the chemotactic chemokines CXCL9 and CXCL10 from these cells (39).

##### NLRP3 Inflammasome Activation

The nucleotide-binding oligomerization domain-like receptor protein 3 (NLRP3) inflammasome is a cytosolic platform that integrates various danger signals into the caspase-1-dependent release of IL-1β and IL-18 (40). TLR9 activation may further contribute to NLRP3 inflammasome assembly in sterile inflammation (41). Allam et al. recently identified histones as NLRP3 inflammasome agonists, which may relate to their particle nature as many crystals and microparticles are NLRP3 agonistic (42). Histones activate NLRP3 mainly through inducing oxidative stress within the cell and induce IL-1 secretion in an NLRP3-ASC-caspase-1-dependent manner. Accordingly, upon intraperitoneal histone injection, NLRP3-deficient mice displayed significantly decreased histone-induced IL-1 production and neutrophil recruitment as a parameter of attenuated histone-induced peritonitis (42).

##### NOD2 Activation

Nucleotide-binding oligomerization domain 2 (NOD2), a member of NOD like receptor family, is involved in histone-originated inflammatory procedures as well (43). High levels of histone H3 were found in LPS-immersed ANA-1 macrophages. The mRNA and protein levels of NOD2, caspase-1, gasdermin D, IL-1β, IL-18 were increased in LPS-stimulated group, which could be reversed by H3 antibodies (43). NLRP3, a downstream factor of NOD2, ran in parallel with NOD2 level and eventually led to pyroptosis (43). Of note, extracellular histones released after pyroptosis have been regarded as one important contributor of serum CHs (11). This vicious cycle denotes the essential role of CHs in the development of sepsis, and confirms CHs’ potential as therapeutic targets in future sepsis management.

##### Complement Activation

Sepsis is associated with the activation of all three complement pathways (classical, alternative and lectin), resulting in the appearance of proinflammatory anaphylatoxins C3a and C5a (28). Bosmann et al. and Kalbitz et al. collectively showed that the interaction between C5a and its receptor (C5aR1 and C5aR2) significantly contributed to the formation of extracellular histones that further mediated acute lung injury and cardiomyopathy in sepsis (29, 44). Neutralization of C5a with antibody or absence of C5aR1 blocks appearance of CHs and alleviates organ failure in sepsis. Meanwhile, histones bind complement C4 to inhibit both classical and mannose-binding lectin pathways dramatically, highlighting a natural feedback mechanism to prevent the excessive injury of host cells (45).

#### Endothelium Injury

Endothelium participates in numerous regulatory functions and contributes to and is affected by inflammatory processes. It is also involved in blood coagulation and fibrinolysis, immune response by modulation of leucocyte interactions with the vessel wall and regulation of vascular tone and blood pressure (46). Endothelium injury in the circulatory system is considered to be the primary cause of sepsis on organ and tissue level (4, 47). It may induce barrier function impair, leakage of plasma-protein-rich fluid into the tissues, alteration of blood flow dynamics, recruitment and activation of circulating leucocytes and release of excessive cytokines (48).

##### Direct Cytotoxicity

Histones may cause direct cytotoxicity to vascular endothelial cells. Administration of high-dose exogenous histone ubiquitously results in a significant reduction in cell viability (49). Current theories suggest that histones bind to phospholipid–phosphodiester bonds, similar to their DNA-binding sites, altering membrane permeability and initiating calcium ion influx (18, 50). Interestingly, C-reactive protein could alleviate histone-mediated toxicity by binding to histones in competition with phospholipid-containing liposomes, blocking histone integration into cell membranes and preventing calcium influx (51).

##### Autophagy and Apoptosis

Autophagy and apoptosis are also found in human umbilical vein endothelial cells (HUVECs) after histone simulation, in which mammalian target of rapamycin (mTOR) plays a significant role (10). When treated with extracellular histones between 10 and 100 μg/mL, Sestrin2 increases and phosphorylates AMPK as well as ULK1, which later immobilizes mTOR. Impaired mTOR activity will then dephosphorylate p70S6K and initiate autophagy procedure (10). Elevated CHs are found to phosphorylate p53 at the same time, resulting in the increase of Bax/Bcl2 ratio and provocation of the apoptotic pathways (10).

##### COX-NOS Interaction

Pérez-Cremades et al. illustrated that endothelium injury was also induced by cyclooxygenases-nitric oxide synthase (COX-NOS) interaction after histone treatment (52). Histones induced prostacyclin (PGI2) production in a dose-dependent manner, decreased thromboxane A2 (TXA2) release and increased a COX-2-dependent superoxide production by upregulating prostacyclin synthase through COX2 synthesis. This change can finally cause a downfall of endothelial NOS and a decrease of nitric oxide production and bioavailability (52).

##### Impaired Cell-Cell Adherens Junction

Meegan et al. further reported that citrullinated histone H3 (H3Cit), one subtype of CHs, showed an ability to thin the adherens junction protein VE-cadherin at borders between cells dramatically and to form an intercellular gap by centralizing F-actin bundles and synthesizing actin stress fiber, causing endothelial barrier dysfunction (53). The impaired barrier would further provide opportunities of invading other sterile tissues and subsequent systematic infectious complications for pathogens.

#### Histones-Induced Disseminated Intravascular Coagulation.

Disseminated intravascular coagulopathy (DIC), characterized by a major dysfunction in hemostasis with increased thrombin generation *in vivo*, is an acquired condition that develops as a complication of systemic and sustained cell injury in sepsis (54). The procoagulant effects of histones, coupled with their ability to kill endothelial cells, activate platelets, and inhibit fibrinolysis, contribute to thrombosis and DIC in sepsis (11, 54–58). In this section, we set out to summary the detailed mechanisms by which histones trigger, amplify and propagate coagulation.

##### Increased Tissue Factor Expression and Reduced Endogenous Anticoagulant Activity

Tissue factor (TF), the initiator of extrinsic pathway, plays a significant role in histone-induced hypercoagulable status during sepsis (35). Recent studies have shown that histones can directly induce TF expression in a dose- and time-dependent manner in vascular endothelial cells and macrophages mediated by the activation of TLR2/4 receptors and the nuclear factor-kappa B (NF-κB) and activator protein 1 (AP-1) pathways (35, 59, 60). When human coronary endothelial cells are treated with of 20 μg/mL of histone, an increase up to 28-fold is found in TF expression. Notably, mutation of the NF-κB and AP-1 binding sites in the TF gene promoter markedly impaired TF promoter activity, especially mutation of NF-κB (35). Kim et al. and Gould et al. also argued that histones simultaneously increased the expression and procoagulant activity of TF in endothelial cells as well as monocytes, and significantly enhanced subsequent thrombin generation that was abrogated with inhibitory antibodies of both TLR2/4 receptors and TF (59–61).

Endogenous anticoagulant pathways—anti-thrombin, protein C, and tissue factor pathway inhibitor—participate in regulating the extent of clot generation (54). Histones significantly induce decreased endothelial surface expression of thrombomodulin and dampen thrombomodulin-dependent protein C activation, which would impair the anticoagulant, anti-inflammatory and cyto-protective functions of activated protein C and thus enhance plasma thrombin generation (59, 62).

##### Atypical Intrinsic Pathway Activation

In physiological terms, the intrinsic pathway of coagulation contributes significantly to increasing thrombin generation and accelerating hemostatic clot formation (54). Sergio and colleagues showed that histone H4 directly bind to prothrombin with an affinity in the low nM range and promoted the generation of thrombin by autoactivation independently of activation of the coagulation cascade (63). Abrams et al. further revealed that histones can directly activate thrombin formation by binding to prothrombin fragments 1 and 2 in the presence of FXa to form a histone-prothrombin-FXa complex while the typical activation of prothrombin requires the formation of factor Va (FVa)-prothrombin-FXa complex (58). Of note, prothrombin activation mediated by histone H3 or H4 is phospholipid-independent, and demands less FXa than the FVa usually does. Likewise, histones also activate the intrinsic pathway through an FXII-dependent mechanism, and histone-DNA complexes significantly contribute to elevated FXII in patients with overt DIC (64). Indirectly, histone-induced release of platelet polyphosphate can stimulate factor XI auto-activation as well as accelerate its thrombin-mediated activation (65).

##### Platelet Activation, Aggregation and Consumption

Platelets play critical roles in histone-mediated intravascular coagulation. Histones bound to platelets, induced calcium influx, and recruited fibrinogen to crosslink platelet integrin αIIbβ3, resulting in subsequent platelet activation, aggregation and consumption *in vitro* and *in vivo* (51, 66–68). At equimolar concentrations, recombinant histone H4 displayed potent activity to induce platelet aggregation, whereas recombinant histones H1, H2A, H2B, and H3, were less effective (67). Histones also induce rapid and profound thrombocytopenia with a prolonged bleeding time in mice, which might be attributed to platelet aggregation in blood. At 50 mg/kg histones, histones deplete ~90% of platelets from circulation within 10 minutes after infusion (67). Likewise, a case-control study also confirmed that high admission histone levels were associated with moderate to severe thrombocytopenia in critically ill patients (69). Notably, histone-induced platelet aggregation could be inhibited by the αIIbβ3 blocker (tirofiban) and anti-histone agents (heparin, albumin, etc.).

Histones also enhance plasma thrombin generation by inducing a procoagulant phenotype of platelets with increased P-selectin expression, phosphatidylserine exposure, and FV/Va availability on platelet surfaces, which is mediated by TLR-2 and TLR-4 (36, 68). P-selectin further enables adherence to the vascular endothelium and leukocytes while augmenting TF expression and phosphatidylserine exposure on monocytes, which collectively enhances thrombin generation (54). Of note, histone-induced TF expression and subsequent thrombin generation can activate platelets in return.

##### Impaired Fibrinolysis

It appears that the controversial effects of histones on the fibrinolytic system are relevant for sepsis-induced DIC. Early studies have consistently revealed that histones could accelerate fibrin polymerization and enhance clot structure rendering it stronger and resistant to fibrinolysis, which could be neutralized by histone-binding low-molecular-weight heparin, or FXIIIa inhibitors (70–72). On one hand, noncovalent histone–fibrin interactions induce lateral aggregation of fibrin protofibrils and increase fibrin-fiber thickness, giving the fibrin network increased mechanical stability. On the other hand, histones also inhibit plasmin as competitive substrates to delay fibrinolysis, and protect fibrin from plasmin digestion through their covalent interactions with fibrin, catalyzed by activated coagulation factor FXIII (FXIIIa) (70). On the contrary, Fabrizio et al. argued that histones strongly accelerated single-chain urokinase-type plasminogen activator (scu-PA)-driven clot lysis through the enhancement of scu-PA to urokinase-type plasminogen activator conversion, mediated by factor seven activating protease (73).

##### Damage or Dysfunction of Endothelial Cells

Damage or dysfunction of endothelial cells is another vital aspect of DIC in sepsis. For instance, histones can stimulate endothelial release of ultra-large *von Willebrand* factor multimers, which are involved in platelet adhesion, platelet consumption, and microvascular thrombosis (74). Likewise, Michels et al. demonstrated that histones induced the release of Weibel–Palade bodies, dynamic endothelial cell organelles that contain procoagulant and proinflammatory mediators including *von Willebrand* factor, in a caspase-dependent, calcium-dependent and charge-dependent manner (75). Meanwhile, membrane disruption of endothelial cells also result in the exposure of highly pro-coagulant negatively charged phospholipid surfaces that can accelerate the prothrombinase reaction by 250,000-fold (76).

In summary, histone significantly contributed to the release of pro-inflammatory cytokines and vascular endothelial injury in sepsis which unfortunately disrupt the fine balance and crosstalk between coagulant, anti-coagulant, and inflammatory pathways to augment the pro-coagulant phenotype (74). First, increased tissue factor expression on endothelial cells and macrophages, pro-inflammatory cytokine release and cellular activation and injury (including platelet activation and endothelium injury) could trigger coagulation in DIC. Second, reduced endogenous anti-coagulant activity, intrinsic pathway activation and impaired fibrinolysis are responsible for the amplification of coagulation in sepsis. Third, the development of MODS further propagates thrombin generation and coagulopathy (54). For a comprehensive understanding of the crosstalk between histone-induced inflammation, endothelium injury and coagulation, a few excellent reviews are recommended to follow (54, 77–79).

### Common Consequences of Histones in Sepsis

As outlined, histones can form vicious cycles to cause distal damage to tissues and organs in sepsis. For example, NETs can be sparked by direct stimulation of citrullinated histone H3 (80). Platelet ballooning can also produce extracellular histones directly, which induce more structure-damaged platelets in return (81). These closed-loops may later lead to DIC, MODS and eventually death. Histones can cause damage to various organs like heart (44, 82), liver (83), lung (84) and kidney (85) by inducing abnormal calcium influx, alteration of genic production, disordered inflammation response, ischemia and hypoxia under sepsis settings. Of note, these signs of tissue injury were usually reduced upon the use of diverse anti-histone agents in *in-vivo* animal experiments.

In summary, histones play an essential role in sepsis development as they are almost involved in each stage of sepsis. It would thus be reasonable to hypothesize that histones have a great potential in serving as diagnostic or prognostic biomarkers for sepsis, and as therapeutic targets.

## Circulating Histones in Sepsis as Potential Diagnostic or Prognostic Biomarkers

Researchers have been studying the diagnostic and prognostic value of CHs in septic patients recently. During their exploration, they gradually shift their attention on confirming the relationship between CHs and certain syndromes to quantitating their accuracy as biomarkers. Pan et al. found that the level circulating H3Cit was significantly higher at 0.5, 3, 12, and 24 hours after LPS injection in LPS-treated mouse group than that in control group, revealing its potential ability as a sensitive and long-lasting biomarker (86). Another observational study also showed an elevated H3Cit level of septic patients, which is far more that of trauma patients (87). The same article confirmed the utility of histones in determining disease progression of sepsis and suggested that H3Cit might serve as an indicator for antibiotics treatment (87). Since then, growing evidences have revealed the significant association between histone levels and heart dysfunction, MODS and death in septic patients.

Cheng et al. performed a receiver operating characteristic (ROC) analysis on 420 critically ill patients, 140 of which were diagnosed with sepsis (16). The ROC analysis demonstrated CHs’ predicting ability of MODS and death by returning an area under the cure (AUC) measuring 0.617 and 0.625 (16). Unfortunately, both cut-off values are not available in this study (16). Consistently, a single-center observational study of 85 septic patients by Yokoyama et al. revealed an AUC of 0.73, suggesting a moderate performance of CHs in predicting morality. The authors also indicated that the predictive performance of circulating histone H3 levels for mortality was higher than that of conventional inflammatory markers, including white blood cell count, C-reactive protein, and cell-free DNA. However, the optical cut-off of CHs was not reported either (88). Kim et al. investigated the levels of NETs in 199 patients with DIC admitted to ICUs and analyzed their potential values to assess coagulation severity and predict clinical outcome. The increased levels of DNA-histone complexes correlated with the severity of coagulopathy including DIC score and D-dimer. The ROC analysis showed an optimal cutoff value of 357 AU and an AUC measuring 0.7 (95%CI 0.629-0.764) (89). It is noteworthy that these findings must be interpreted with great caution as only 31 out of 199 patients had sepsis. More recently, in a prospective observational study on 126 septic patients, Lu et al. found that high plasma histone H4 levels were independent risk factors for predicting mortality using a multivariate logistic analysis and that plasma histone H4 level positively correlated with sequential organ failure assessment (SOFA) score, cardiac troponin, N-terminal pro-B-type natriuretic peptide, and lactate levels (90). Further ROC curve analysis showed that the sensitivity and specificity of either plasma histone level or SOFA score were highly predictive of mortality, but the predictive effect of histone H4 was more substantial than SOFA score. Plasma histone H4 significantly predicted mortality with a sensitivity of 70.3%, a specificity of 76.4%, an AUC of 0.731 when the cutoff point was set as 0.30 µg/mL. On the contrary, the prediction of death had a sensitivity of 73%, a specificity of 58.4% and an AUC of 0.67 when the cutoff point was set as 9.5 for SOFA score (90).

We may notice that the results of the above-mentioned articles do not present a very favorable outcome confirming the diagnostic or prognostic value of CHs as there is still no gold-standard detection method to determine the concentration of CHs in septic patients. The level of CHs is mainly determined by western blotting quantification and enzyme-linked immunosorbent assay at present (54). Immunoassays usually bear many flaws including poor concordance between assays, differences in reproducibility and variable cross-reactivity (91). Meanwhile, different kits used in these clinical trials may influence the study results and therefore do harm to the external validity of these trials. Tandem mass spectrometry may represent a detection method capable of alleviating many of the flaws inherent to immunoassays and thus should be used to increase the sensitivity and specificity of histone detection (91, 92). For instance, García-Giménez and colleagues proposed a novel method to detect histone H3 and H2B in plasma from septic patients based on a targeted, quantitative multiple reaction monitoring targeted mass spectrometry (MRM-MS), which allows histone quantification using an internal standard with high specificity and sensitivity (92). Generally, the authors first selected peptides LLLPGELAK and STELLIR that respectively derived from the tryptic digestion of purified H2B and H3 as unique spiked-in labeled proteotypic peptides. Then, these peptide sequences were used to spike the plasma samples and the levels of histones H2B and H3 were quantified in plasma samples by the MRM-MS approach using standard curves with different concentrations of commercial histone H2B and H3 and stable isotopically labelled peptides (92). By the MRM-MS approach, histone H3 can significantly distinguish septic shock cases from healthy controls, with a sensitivity of 94.1%, a specificity of 90.0%, and an AUC of 0.982 when a concentration of 574.25 ng/mL was taken as the optimal cut-off value (92).

Another difficulty facing the implementation of such biomarkers is that histones also mediate other diseases such as stroke, trauma, diabetic retinopathy, cancer, and rheumatoid arthritis, and an elevation level of CHs has also been found in these syndromes, how to interpret the test result in clinical settings would be of great importance and complexity (11, 93, 94).

## Circulating Histones in Sepsis as Potential Therapeutic Targets

CHs are major mediators of death in sepsis by inducing neutrophil margination, vacuolated endothelium, intra-alveolar hemorrhage and macro- and microvascular thrombosis (50). However, their potential to amplify tissue injury by killing other cells in addition to their agonistic activity on TLRs and the NLRP3 inflammasome provides a rationale to target CHs for therapy. The aim of this chapter is to summary the latest literatures concerning the development of novel histone blocking agents to neutralize the cytotoxic effect of positively charged CHs, thereby potentially reducing organ damage and mortality.

### Heparins, Non-Anticoagulant Heparins and Heparinoids

Heparin, a negatively charged glycosaminoglycan derived from porcine intestine, has been used as an anticoagulant for decades (95). Beyond anticoagulation, heparin inhibits the function of several adhesion molecules, the synthesis of inflammatory cytokines including TNF-α, IFN-γ, IL-6, and IL-8 *via* inhibition of NF-κB signaling, and the cleavage of complement proteins (96). Usually, the mechanisms by which heparin is able to express its anti-inflammatory properties can be divided into two models of action: 1) binding to soluble plasma ligands such as complement proteins, pro-inflammatory chemokines and cytokines, which significantly interferes the classical and alternative complement pathways as well as terminal cell lysis and dissociates cytokines and other proteins from their stationary binding; 2) binding to cell-surface-bound receptors or macromolecules including P-selectin, L-selectin, and intercellular adhesion molecule-1, which inhibits interactions between endothelial cells and blood cells (e.g., leukocytes, platelets) (97). Of note, these functions are mostly affected by structural parameters, such as chain length, degree of sulfation, carbohydrate backbone and sulfation pattern (95). Several recently published meta-analyses demonstrated that the use of unfractionated heparin (UFH) or low-molecule-weight heparin (LMWH) resulted in decreased 28-day mortality in sepsis despite increased bleeding events were recorded (98–100). As septic patients often have a compromised coagulation system, the occurrence of bleeding prevents a more widespread clinical use of heparin as an anti-inflammatory agent in this particular population.

#### Heparin

Growing evidences have further suggested that heparin effectively binds to positively-charged histones through high affinity electrostatic interactions and form heparin-histone complexes which are devoid of cytotoxicity (101–103). Longstaff et al. revealed that the apparent equilibrium dissociation constants of UFH, LMWH and enoxaparin for histone binding were 0.4, 0.8 and 0.5 µg/ml, respectively, using a surface plasmon resonance approach (103). These heparins were effective at displacing DNA from the immobilized histones. Of note, this particular interaction between histones and heparins also contributes to the neutralization of anticoagulant effects of heparins (103). Recently, heparin and its derivatives have been showed to attenuate histone-mediated cytotoxicity, coagulation and inflammation in a dose-dependent manner, presumably by binding to histones to disturb the interactions between histones and TLRs, platelets, neutrophils, endothelial cells etc. Zhu et al. further revealed that heparin administration reduced histone-induced endothelial damage and coagulation activation with decreased von Willebrand factor and soluble thrombomodulin, thus prevented intestinal microcirculatory dysfunction in histone-infused rats (102). They claimed that calcium influx, instead of the release of intracellular Ca^2+^, was a major mechanism explaining histones toxicity and heparin inhibited calcium influx almost entirely. Similarly, Wang et al. found that co-injection of low dose UFH (10 mg/kg) with lethal dose histones (75 mg/kg) could protect septic mice from organ damage and death by antagonizing histones (104). On the contrary, histone-infused mice treated by argatroban, another small molecule anti-coagulant directly inhibiting thrombin, failed to survive, suggesting that the protective effect of heparin mainly depended on its direct neutralization to histones rather than anticoagulation (105). Meanwhile, UFH administration could also attenuate histone-induced inflammatory response and complement activation in whole blood *in vitro*. Concentrations of generated IL-6, IL-8, C3a and C5a significantly reduced in a dose-dependent or –independent manner when UFH was added to histone-induced whole blood (106). It is noteworthy that heparin is usually pretreated or injected with histone in above-mentioned *in vitro* or *in vivo* studies, which is obviously inconsistent with clinical practice. In these experimental settings, the protective effects of UFH might be exaggerated. Therefore, *in vivo* animal experiments were conducted by Li et al. to confirm whether heparin intervention was still valid when histones already resulted in tissue damage. In this study, 400 U/kg of UFH was intentionally injected into C57BL/6 mice through the tail vein 1 h or 6 h after the induction of histones. UFH was shown to improve survival rate in mice injected with lethal doses of histones and alleviate histone-induced lung injury, pulmonary edema, endothelial cell injury, coagulation activation and thrombosis in a dose-dependent manner (107). Likewise, Wang et al. revealed that a subcutaneous injection of unfractionated heparin 4 h after cecal ligation and puncture operation (CLP) significantly improved the 72-h survival rate of CLP-treated C57BL/6 mice, decreased serum levels of histone H4, neutrophil gelatinase-associated lipocalin, kidney injury molecule-1, TNF-α, and IL-6, alleviated kidney tissue edema and apoptosis, and thus protected septic mice from acute kidney injury (108). These results theoretically support the precise use of UFH in patients with sepsis although the risk of hemorrhage related to relatively high doses of heparin is still of great concern due to the presence of coagulation disorders in these patients.

#### Non-Anticoagulant Heparins

As outlined, anti-inflammatory effect of heparin is independent of its anticoagulant activity. On the contrary, the bleeding side effect associated with anticoagulation can be an inherent drawback for heparin to act as an anti-inflammatory agent. Hogwood et al. found that the anti-inflammatory effect of heparin significantly depended on its sulfated level. Partially-selectively-desulfated heparin with reduced anticoagulant activity maintained an anti-inflammatory property in whole blood while complete sulfate removal rendered heparin unable to act as an anti-histone agent, demonstrating the importance of negatively charged sulfate groups (106). Modification of heparin to reduce or remove anticoagulant activity is thus an attractive approach to improve its therapeutic potential in this particular setting. For example, Wildhagen et al. developed an anti-thrombin affinity depleted heparin (AADH) by removal of anticoagulant fraction of UFH (101). Surface plasmon resonance and cell culture experiments showed that AADH directly bound to histones with an apparent dissociation constant of 86 nM and could effectively block histone-mediated cytotoxicity. A significant improvement of survival in both CLP-induced and LPS-induced septic murine models treated with 570 μg of AADH was also observed. AADH administration appeared to decrease neutrophil influx, protected against intrapulmonary protein leakage and capillary-alveolar leakage, and was accompanied by reduction of cytokine release. Meanwhile, AADH treatment of unchallenged mice with a dose 5 times that of UFH caused only moderate anticoagulation of blood plasma and no significant prolongation of tail bleeding time.

#### Heparinoids

Rasmuson et al. revealed that sevuparin, a heparinoid with low anticoagulant activity, prevented neutrophil-induced lung plasma leakage in a murine model of systemic inflammation evoked by heat-killed group A *Streptococcus* (109). Their results demonstrated that sevuparin could interact with neutrophil secretion including serprocidins, S100 proteins and histone H4 and thus attenuate the disruptive effect of histone on endothelial integrity, suggesting a potential therapeutic of sevuparin in acute neutrophilic inflammation like sepsis.

More recently, Li et al. developed an efficient approach to synthesize homogeneous chondroitin sulfate E (CS-E) oligosaccharides, another type of heparinoids which could bind to extracellular histones directly, and investigated their anti-inflammatory effects (110). They found that CS-E treatment could neutralize the cytotoxic effect of histones in a cell-based assay and in mice in a dose-dependent manner. The optimized CS-E 19-mer was the tightest binder to histone H3 with a binding affinity of 44.7 nM among the developed CS-E compounds. Treatment with CS-E 19-mer attenuated LPS-induced lung vascular permeability as measured by leakage of Evans Blue into lung tissue, indicating protection against endothelial cell damage. Most importantly, they found that CS-E 19-mer treatment significantly reduced the LPS-induced mortality rate of septic mice from 92% to 30%, with improved kidney and liver functions. Of note, CS-E 19-mer did not contain anticoagulant activity as demonstrated by the absence of anti-FXa and anti-FIIa activities, suggesting its certain advantages over heparin and heparan sulfate by eliminating the bleeding concern of heparin use in septic patients.

### Recombinant Thrombomodulin and Activated Protein C

The thrombomodulin (TM)/activated protein C (APC) system plays an important role in maintaining the homeostasis of thrombosis and hemostasis and maintaining vascular integrity *in vivo* (111). TM is mainly expressed on the endothelium of blood vessels, which can bind to thrombin and act as a vital suppressor of intravascular coagulation. Previous studies have already revealed the anti-inflammatory and cyto-protective actions of TM, which is preserved in its lectin-like domain (66, 112–114). The lectin-like domain of TM binds high-mobility group box 1 protein and inhibits its signaling *via* blocking the receptor for advanced glycation end products, which could apparently improve the survival of LPS-challenged mice (115). Intriguingly, TM also directly binds and inactivates histones (62, 66). The negatively charged domains of TM, the O-linked chondroitin sulfate glycosaminoglycan (GAG) moiety, are likely to interact with cationic proteins including histones. TM was shown to reduce histone-induced platelet aggregation and protect mice from lethal thromboembolism (66). Nakahar et al. demonstrated that administration of recombinant TM significantly reduced histone H3 levels and improved kidney injury. The survival rate of rats in recombinant TM-treated group was also significantly higher than that in the vehicle-treated group (40% vs. 20%, P = 0.048) (113).

APC is a multifunctional seine protease with anticoagulant, cyto-protective and anti-inflammatory property (111). In physiological conditions, APC inactivates factors VIIIa and Va in the presence of protein S, thereby inhibiting further thrombin formation. APC can also cleave G protein-coupled receptor protease-activated receptor-1 (PAR1) at Arg46 and mediate a downstream signal transduction pathway, leading to the generation of anti-inflammatory as well as cyto-protective activities (116). APC binds to DAMPs, histone H3, and H4 through its densely anionic N-terminal Gla domain by means of electrostatic forces and subsequently cleaves these histones in a PAR1-independent manner, implying a possible link between APC function and NETs formation (62, 117, 118). In a murine LPS-induced sepsis model, APC administration resulted in a significant decrease in histone H3 and H4 level, and effectively blocked the cytotoxicity of histones (50). Meanwhile, concomitant use of APC rescued all mice challenged with a lethal dose of histones. Notably, this anti-histone activity may be one of the most attractive functions of APC to rescue septic DIC patients, as significantly higher plasma levels of histone H3 were observed in non-survivors with septic DIC compared with survivors (66).

### Monoclonal Antibody

After being released from ruptured neutrophils, histones bind to TLRs and initiate a vicious cycle, resulting in propagation of inflammatory response. As such, researchers investigated the utility of anti-H3Cit antibody in sepsis, whose results turned to be remarkable (119, 120). Deng et al. developed a novel anti-H3Cit (citrullinated R2+R8+R17+R26) monoclonal antibody [H3Cit mAb (4 Cit)] (120). This novel antibody was able to diminish serum level of cf-DNA and serum cytokines including IL-1β and TNF-α, thus improving survival in LPS-induced endotoxemia mice (120). Of note, compared with another anti-H3Cit antibody (citrullinated R2+R8+R17), H3Cit mAb (4 Cit) showed better histone binding capacity and a survival benefit, demonstrating its utility as an anti-histone agent against septic shock (120). Besides, Xu et al. demonstrated antibody to histone H4 significantly lower the mortality of mice in both LPS- and CLP-induced septic models *in vivo*. In contrast, the antibody to histone H2B failed to rescue the mice (50). However, safety evaluation on antibodies to histones antibodies in septic models is absent at present. Solid data from *in vivo* animal experiments are thus needed to support the use of these antibodies to histones for sepsis treatment.

### Polysialic Acid

Ploysialic acid (PSA), composed of alpha-2,8-linked N-acetylneuranminic acid residues, is a negatively charged carbohydrate in human plasma and different branches of vertebrates (121). Recently, PSA has been considered as a novel candidate to combat excessive NETosis due to its efficacy in antagonizing cytotoxicity of histones (122–125). Mishra et al. identified that PSA could directly bind to histone H1 *via* a single-chain variable fragment antibody using an anti-idiotypic approach (122). Then, Saffarzadeh et al. found that pre-incubation of histones or NET with PSA considerably reduced both histone- and NET-mediated cytotoxicity *via* the electrostatic interaction between PSA and histones (123). This hypothesis was further supported by two later publications (124, 125). Intriguingly, PSA binds histones and reduces histone-mediated cytotoxicity in a chain length-dependent manner. PSA chains originating from human plasma consisted of more than 40 sialic acid residues and showed a cyto-protective effect against extracellular histones (125). Recently, Galuska et al. fabricated an artificial polysialic chain by oxidizing the terminal sialic acid residue. While maintaining the ability to antagonize histone-mediated cytotoxicity, the transformation of the non-reducing end enabled resulting PSA chains to accumulate nanoparticles on NET-fibers (126). These bound particles were loaded with active substances, which could be utilized for biomedical application (126). Later, polysialylated mucins were developed by elongating cervical mucins with bacterial polysialyltransferases, which could attenuate the adhesion of neutrophils and decrease histone-mediated cytotoxicity (127). Of note, the efficacy and safety of PSA or its derivatives has not been evaluated in *in vivo* histone-induced animal models so far and solid evidences are urgently required to confirm the feasibility of using such compounds in patients with sepsis.

## Conclusion and Outlooks

Once being released from necrotic cells, CHs exhibit toxic effects not only on pathogens but also on host cells. They initiate and amplify dysregulated inflammatory response by activating TLRs and the NLRP3 inflammasome, and induce endothelium injury as well as coagulation activation, which consequently lead to MODS and death. Clinical studies have also confirmed the close correlation between high levels of CHs and disease severity as well as mortality in sepsis, suggesting that CHs have the potential to predict the clinical outcome of sepsis. Besides, histone-related immunopathology can be targeted by inactivating histones with anti-histone agents including heparin, recombinant thrombomodulin, activated protein C, monoclonal antibody, and polysialic acid. Non-anticoagulant anti-histone agents such as modified heparin derivatives and polysialic acid also exhibit certain advantages over unfractionated heparin, thrombomodulin and activated protein C by lowering bleeding risks in patients with sepsis and DIC.

Of note, the immunopathological role of histones in initiating or propagating inflammation, mediating endothelial damage and activating coagulation system is not completely understood. Precise mechanisms of histone-induced endothelial permeability and cytotoxicity await further characterization due to complexity and diversity of mechanisms regulating endothelial barrier function. Meanwhile, accurate detection methods for CH are still of great need to monitor the histone levels for outcome prediction in septic patients. Conventional ELISA and Western blotting techniques to measure histone levels in patient samples so far need to be replaced with more sensitive and real-time monitoring assays such as detection by probes *in situ*. A careful analysis of histone subtypes as well as histone modifications (namely citrullinated histone) also needs to be done because of the significant variation in their toxicity (124, 128). Another major area of investigation is on the discovery of novel anti-histone agents to eliminate the unexpected bleeding risk associated with the use of conventional heparin and thrombomodulin, etc. Like heparin and polysialic acid, natural anion polysaccharides such as alginic acid, and pectin also have the potential to bind histone directly *in vitro* (129). Given their negligible effect on coagulation system, further studies are required to determine whether the intravenous use of these polysaccharides could alleviate histone-induced pathologies in sepsis models to highlight their non-anticoagulant properties as an underlying advantage for anti-histone therapy. To explore whether extracorporeal blood purification procedures using heparin-grafted hemofilters such as AN69 ST and oXiris membrane, which have been widely used in patients with severe sepsis for cytokine removal, could lower the elevated histone level in blood circulation and improve the survival of this particular population would be also valuable.

## Author Contributions

YL, DW, and XL drafted the manuscript and have contributed equally to this work. All authors contributed to the article and approved the submitted version.

## Funding

We acknowledge that this work is financially sponsored by the State Key Research Programme of China (Nos. 2016YFC1103003 and Nos. 2016YFC1103004), the National Natural Science Foundation of China (Nos. 82000702), and the Science and Technology Achievement Transformation Fund of West China Hospital of Sichuan University (Nos. CGZH19006).

## Conflict of Interest

The authors declare that the research was conducted in the absence of any commercial or financial relationships that could be construed as a potential conflict of interest.
